# 5-Amino-5′-bromo-6-(4-methyl­benzo­yl)-8-nitro-2,3-di­hydro-1*H*-spiro­[imidazo[1,2-*a*]pyridine-7,3′-indolin]-2′-one including an unknown solvate

**DOI:** 10.1107/S1600536814014391

**Published:** 2014-06-25

**Authors:** R. A. Nagalakshmi, J. Suresh, S. Sivakumar, R. Ranjith Kumar, P. L. Nilantha Lakshman

**Affiliations:** a Department of Physics, The Madura College, Madurai 625 011, India; b Department of Organic Chemistry, School of Chemistry, Madurai Kamaraj University, Madurai 625 021, India; c Department of Food Science and Technology, University of Ruhuna, Mapalana, Kamburupitiya 81100, Sri Lanka

**Keywords:** crystal structure

## Abstract

In the title compound, C_22_H_18_BrN_5_O_4_, the central six-membered ring, derived from 1,4-di­hydro­pyridine, adopts a distorted boat conformation with a puckering amplitude of 0.197 (3) Å, the imidazole ring adopts a twisted conformation with a puckering amplitude of 0.113 (3) Å, and the oxindole moiety is planar with an r.m.s. deviation of 0.0125 Å. Two intra­molecular N—H⋯O hydrogen bonds are formed, each closing an *S*(6) loop. In the crystal, strong N—H⋯O hydrogen bonds lead to the formation of zigzag chains along the *c* axis. These are consolidated in the three-dimensional crystal packing by weak N—H⋯O hydrogen bonding, as well as by C—H⋯O, C—H⋯Br and C—H⋯π inter­actions. A small region of electron density well removed from the main mol­ecule was removed with the SQUEEZE procedure in *PLATON* [Spek (2009 ▶). *Acta Cryst*. D**65**, 148–155] following unsuccessful attempts to model it as a plausible solvent mol­ecule. The unit-cell characteristics do not take into account this feature of the structure.

## Related literature   

For a similar structure, see: Nagalakshmi *et al.* (2014 ▶). For additional conformational analysis, see: Cremer & Pople (1975 ▶).
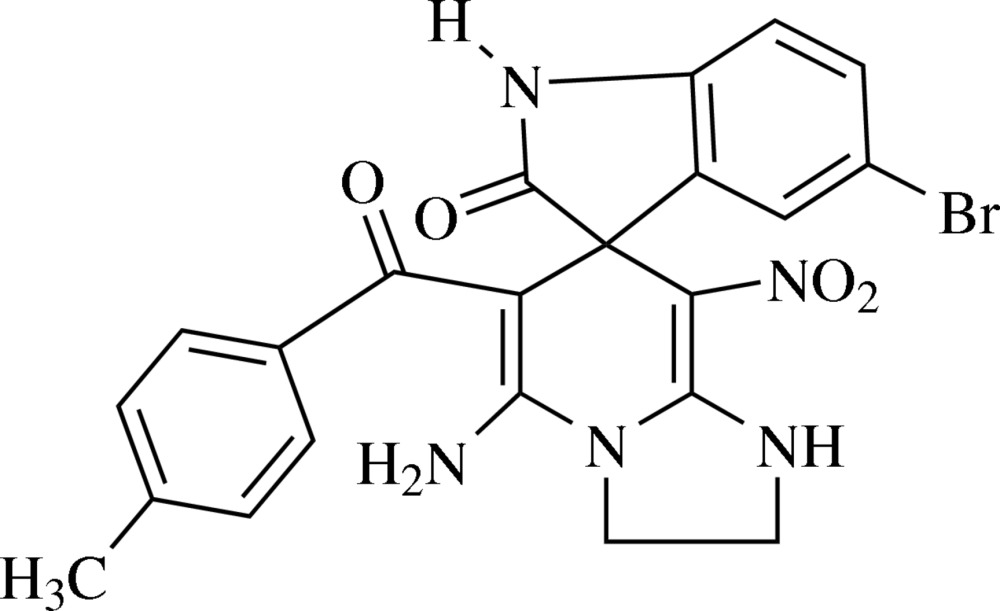


## Experimental   

### 

#### Crystal data  

C_22_H_18_BrN_5_O_4_
*M* *_r_* = 496.32
Monoclinic, 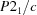

*a* = 15.5482 (9) Å
*b* = 14.7033 (7) Å
*c* = 12.1907 (6) Å
β = 101.856 (2)°
*V* = 2727.5 (2) Å^3^
*Z* = 4
Mo *K*α radiation
μ = 1.54 mm^−1^
*T* = 293 K
0.21 × 0.19 × 0.18 mm

#### Data collection  

Bruker Kappa APEXII diffractometer
Absorption correction: multi-scan (*SADABS*; Bruker, 2004 ▶) *T*_min_ = 0.967, *T*_max_ = 0.974
30073 measured reflections
5962 independent reflections
4098 reflections with *I* > 2σ(*I*)
*R*_int_ = 0.035

#### Refinement  

*R*[*F* ^2^ > 2σ(*F* ^2^)] = 0.040
*wR*(*F* ^2^) = 0.114
*S* = 1.04
5962 reflections
289 parameters
1 restraint
H-atom parameters constrained
Δρ_max_ = 0.30 e Å^−3^
Δρ_min_ = −0.41 e Å^−3^

### 

Data collection: *APEX2* (Bruker, 2004 ▶); cell refinement: *SAINT* (Bruker, 2004 ▶); data reduction: *SAINT*; program(s) used to solve structure: *SHELXS97* (Sheldrick, 2008 ▶); program(s) used to refine structure: *SHELXL97* (Sheldrick, 2008 ▶); molecular graphics: *PLATON* (Spek, 2009 ▶); software used to prepare material for publication: *SHELXL97*.

## Supplementary Material

Crystal structure: contains datablock(s) global, I. DOI: 10.1107/S1600536814014391/tk5316sup1.cif

Structure factors: contains datablock(s) I. DOI: 10.1107/S1600536814014391/tk5316Isup2.hkl

Supporting information file. DOI: 10.1107/S1600536814014391/tk5316Isup3.cml

CCDC reference: 1009067

Additional supporting information:  crystallographic information; 3D view; checkCIF report

## Figures and Tables

**Table 1:** Hydrogen-bond geometry (Å, °) *Cg*1 is the centroid of the C32–C37 ring.

*D*—H⋯*A*	*D*—H	H⋯*A*	*D*⋯*A*	*D*—H⋯*A*
N5—H5⋯O1	0.86	2.09	2.608 (2)	118
N2—H2*B*⋯O4	0.86	1.87	2.518 (2)	131
N3—H3⋯O4^i^	0.86	1.95	2.792 (2)	168
N5—H5⋯O3^ii^	0.86	2.36	2.961 (2)	127
C7—H7*A*⋯O3^iii^	0.97	2.54	3.342 (3)	140
C33—H33⋯Br1^iv^	0.93	2.91	3.675 (2)	141
C14—H14⋯*Cg*1^i^	0.93	2.83	3.553 (2)	135

Symmetry codes: (i) 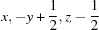; (ii) 
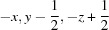
; (iii) 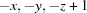; (iv) 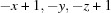.

**Fig. 1.**
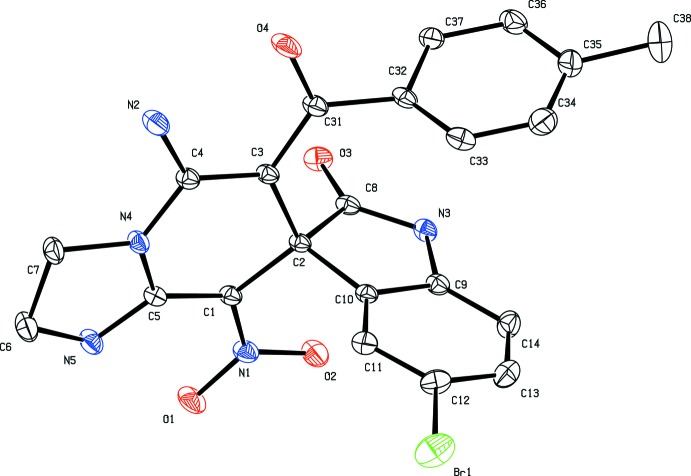
The molecular structure of (I), showing 20% probability displacement ellipsoids and the atom-numbering scheme. H-atoms are omitted for clarity.

**Fig. 2.**
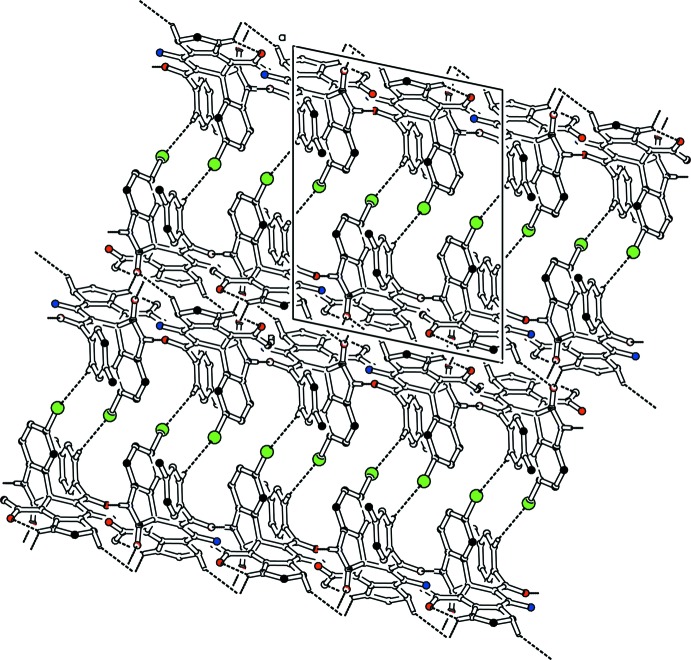
Partial packing diagram of the title compound. Dashed bonds represent inter-molecular hydrogen bonds.

## supplementary crystallographic information

### S2. Structural commentary

Our inter­est in preparing pharmacologically active pyridine-related compounds (Nagalakshmi *et al.*, 2014) led us to the title compound, derived from a 1,4-di­hydro­pyridine. We have undertaken an X-ray crystal structure determination of this compound in order to establish its molecular conformation.

In the title compound (Fig. 1), the central six-membered ring adopts a distorted-boat conformation with the puckering parameters Q = 0.197 (3) Å and θ = 102.0 (10)° and φ = 9.1 (6)° (Cremer & Pople, 1975). The imidazole ring adopts a twisted conformation with puckering parameters Q = 0.113 (3) Å and φ_2_ = 302.9 (11)° (Cremer & Pople, 1975). The oxindole moiety (C2/C8—C14/N3/O3) is planar with r.m.s. deviation of 0.0125 Å. The sum of valence angles at N2 (360 (3)°) indicates that the atom N2 is sp^2^ hybridized. There is a partial delocalization of the lone pair of N2 towards the pyridine ring which is confirmed by the short bond length of C4–N2 = 1.324 (3) Å. The C–N and C–C bond lengths (C4–N4 = 1.361 (3) Å, N4–C5 = 1.365 (3) Å, C1–C2 = 1.523 (3) Å are shorter than the standard C–N = 1.47 Å and C–C = 1.54 Å, respectively. By contrast, the C═C bond lengths (C1═C5 = 1.383 (3) Å and C4═C3 = 1.409 (3) Å) are longer than the standard C═C bond (1.34 Å). Thus, the title compound shows that there is a homo-conjugation effect on the pyridine moiety.

In the crystal, N3—H3···O4 hydrogen bonds lead to the formation of chains along the c axis. N5—H5···O3 hydrogen bonds lead to the formation of chains along the b axis. There are further C7—H7A···O3 and C33—H33···Br1 hydrogen bonds enclosing *R*_2_^2^(16) and *R*_2_^2^(20) ring motifs respectively as shown in Fig. 2. The structure is further stabilized by weak C—H···π inter-molecular inter­actions.

### S5. Synthesis and crystallization

A mixture of 4-methyl­benzoyl­aceto­nitrile (1.0 mmol), 5-bromo­isatin (1.0 mmol) and 2-(nitro­methyl­ene)imidazolidine were dissolved in 10 ml of EtOH and tri­ethyl­amine (1.0 mmol) was added and the reaction mixture was heated to reflux for 45 min. After completion of the reaction, as evident from TLC, the precipitated solid product was filtered and dried to obtain pure pale brown solid. Yield 91 %. Melting point 530 K.

### S6. Refinement

H atoms were placed in calculated positions and allowed to ride on their carrier atoms with C—H = 0.93 (aromatic CH), 0.96 (methyl CH_3_) or 0.97 Å (methyl­ene CH_2_), and N—H = 0.86 Å. Isotropic displacement parameters for H atoms were calculated as *U*_iso_ = 1.5*U*_eq_(C) for CH_3_ groups and *U*_iso_ = 1.2*U*_eq_(carrier atom) for all other H atoms. A small region of electron density well removed from the main molecule and appearing disordered was removed with PLATON SQUEEZE [Spek (2009). *Acta Cryst*. D65, 148–155] following unsuccessful attempts to model it as plausible solvent molecule.

### Crystal data

**Table:**

C_22_H_18_BrN_5_O_4_	*F*(000) = 1008
*M**_r_* = 496.32	*D*_x_ = 1.209 Mg m^−^^3^
Monoclinic, *P*2_1_/*c*	Melting point: 530 K
Hall symbol: -P 2ybc	Mo *K*α radiation, λ = 0.71073 Å
*a* = 15.5482 (9) Å	Cell parameters from 2000 reflections
*b* = 14.7033 (7) Å	θ = 2–31°
*c* = 12.1907 (6) Å	µ = 1.54 mm^−^^1^
β = 101.856 (2)°	*T* = 293 K
*V* = 2727.5 (2) Å^3^	Block, brown
*Z* = 4	0.21 × 0.19 × 0.18 mm

### Data collection

**Table:**

Bruker Kappa APEXII diffractometer	5962 independent reflections
Radiation source: fine-focus sealed tube	4098 reflections with *I* > 2σ(*I*)
Graphite monochromator	*R*_int_ = 0.035
Detector resolution: 0 pixels mm^-1^	θ_max_ = 27.0°, θ_min_ = 1.9°
ω and φ scans	*h* = −19→11
Absorption correction: multi-scan (*SADABS*; Bruker, 2004)	*k* = −18→18
*T*_min_ = 0.967, *T*_max_ = 0.974	*l* = −15→15
30073 measured reflections	

### Refinement

**Table:**

Refinement on *F*^2^	Primary atom site location: structure-invariant direct methods
Least-squares matrix: full	Secondary atom site location: difference Fourier map
*R*[*F*^2^ > 2σ(*F*^2^)] = 0.040	Hydrogen site location: inferred from neighbouring sites
*wR*(*F*^2^) = 0.114	H-atom parameters constrained
*S* = 1.04	*w* = 1/[σ^2^(*F*_o_^2^) + (0.0653*P*)^2^] where *P* = (*F*_o_^2^ + 2*F*_c_^2^)/3
5962 reflections	(Δ/σ)_max_ < 0.001
289 parameters	Δρ_max_ = 0.30 e Å^−^^3^
1 restraint	Δρ_min_ = −0.41 e Å^−^^3^

### Special details

**Table:**

Geometry. All e.s.d.'s (except the e.s.d. in the dihedral angle between two l.s. planes) are estimated using the full covariance matrix. The cell e.s.d.'s are taken into account individually in the estimation of e.s.d.'s in distances, angles and torsion angles; correlations between e.s.d.'s in cell parameters are only used when they are defined by crystal symmetry. An approximate (isotropic) treatment of cell e.s.d.'s is used for estimating e.s.d.'s involving l.s. planes.
Refinement. Refinement of *F*^2^ against ALL reflections. The weighted *R*-factor *wR* and goodness of fit *S* are based on *F*^2^, conventional *R*-factors *R* are based on *F*, with *F* set to zero for negative *F*^2^. The threshold expression of *F*^2^ > σ(*F*^2^) is used only for calculating *R*-factors(gt) *etc*. and is not relevant to the choice of reflections for refinement. *R*-factors based on *F*^2^ are statistically about twice as large as those based on *F*, and *R*-factors based on ALL data will be even larger.

### Fractional atomic coordinates and isotropic or equivalent isotropic displacement parameters (Å^2^)

**Table:**

	*x*	*y*	*z*	*U*_iso_*/*U*_eq_	
C1	0.10831 (12)	−0.03982 (13)	0.29919 (16)	0.0302 (4)	
C2	0.16209 (12)	0.04483 (12)	0.33939 (15)	0.0273 (4)	
C3	0.15943 (12)	0.06548 (13)	0.46280 (15)	0.0302 (4)	
C4	0.13013 (12)	−0.00186 (13)	0.52880 (16)	0.0335 (4)	
C5	0.08565 (12)	−0.10239 (13)	0.37313 (16)	0.0315 (4)	
C6	0.04187 (16)	−0.22940 (16)	0.45722 (19)	0.0509 (6)	
H6A	0.0818	−0.2808	0.4694	0.061*	
H6B	−0.0174	−0.2508	0.4555	0.061*	
C7	0.06850 (15)	−0.15800 (14)	0.54656 (19)	0.0421 (5)	
H7A	0.0184	−0.1374	0.5759	0.051*	
H7B	0.1133	−0.1808	0.6078	0.051*	
C8	0.12622 (12)	0.12869 (13)	0.26493 (15)	0.0305 (4)	
C9	0.26599 (13)	0.10750 (13)	0.24292 (16)	0.0327 (4)	
C10	0.25462 (12)	0.03862 (12)	0.31650 (15)	0.0291 (4)	
C11	0.32166 (12)	−0.02155 (14)	0.35672 (16)	0.0363 (5)	
H11	0.3146	−0.0680	0.4059	0.044*	
C12	0.39967 (14)	−0.01014 (17)	0.32108 (19)	0.0461 (5)	
C13	0.41184 (14)	0.05743 (17)	0.2473 (2)	0.0498 (6)	
H13	0.4653	0.0626	0.2250	0.060*	
C14	0.34405 (14)	0.11796 (16)	0.20630 (18)	0.0449 (5)	
H14	0.3509	0.1637	0.1562	0.054*	
C31	0.18598 (14)	0.15050 (13)	0.51348 (17)	0.0376 (5)	
C32	0.23171 (13)	0.22376 (13)	0.46127 (16)	0.0346 (4)	
C33	0.31931 (14)	0.21652 (15)	0.45811 (19)	0.0452 (5)	
H33	0.3489	0.1621	0.4781	0.054*	
C34	0.36363 (16)	0.29024 (19)	0.4251 (2)	0.0608 (6)	
H34	0.4228	0.2845	0.4230	0.073*	
C35	0.3216 (2)	0.37187 (19)	0.3955 (2)	0.0621 (7)	
C36	0.23388 (18)	0.37875 (16)	0.3996 (2)	0.0515 (6)	
H36	0.2043	0.4332	0.3798	0.062*	
C37	0.18933 (14)	0.30590 (14)	0.43283 (16)	0.0381 (5)	
H37	0.1304	0.3120	0.4361	0.046*	
C38	0.3699 (3)	0.4543 (3)	0.3619 (4)	0.1206 (16)	
H38A	0.3299	0.5046	0.3458	0.181*	
H38B	0.3926	0.4399	0.2965	0.181*	
H38C	0.4175	0.4703	0.4223	0.181*	
N1	0.09080 (11)	−0.06034 (12)	0.18652 (14)	0.0389 (4)	
N2	0.12708 (14)	0.00993 (13)	0.63557 (15)	0.0548 (5)	
H2A	0.1089	−0.0333	0.6725	0.066*	
H2B	0.1433	0.0608	0.6680	0.066*	
N3	0.19037 (10)	0.16040 (11)	0.21672 (13)	0.0336 (4)	
H3	0.1854	0.2078	0.1746	0.040*	
N4	0.10306 (11)	−0.08480 (10)	0.48544 (13)	0.0342 (4)	
N5	0.04712 (11)	−0.18207 (12)	0.35518 (15)	0.0412 (4)	
H5	0.0271	−0.2038	0.2894	0.049*	
O1	0.05241 (12)	−0.13281 (11)	0.15117 (13)	0.0546 (4)	
O2	0.11477 (11)	−0.00570 (11)	0.12016 (12)	0.0494 (4)	
O3	0.05189 (9)	0.15950 (10)	0.25570 (12)	0.0413 (4)	
O4	0.17505 (15)	0.17071 (11)	0.60983 (14)	0.0669 (5)	
Br1	0.493532 (17)	−0.09216 (2)	0.37527 (3)	0.07818 (15)	

### Atomic displacement parameters (Å^2^)

**Table:**

	*U*^11^	*U*^22^	*U*^33^	*U*^12^	*U*^13^	*U*^23^
C1	0.0390 (9)	0.0255 (10)	0.0254 (10)	0.0006 (7)	0.0052 (8)	−0.0020 (8)
C2	0.0378 (9)	0.0216 (10)	0.0219 (9)	0.0030 (7)	0.0048 (7)	0.0008 (8)
C3	0.0428 (10)	0.0230 (10)	0.0243 (10)	0.0030 (8)	0.0058 (8)	0.0009 (8)
C4	0.0427 (10)	0.0283 (11)	0.0290 (11)	0.0032 (8)	0.0059 (8)	0.0012 (9)
C5	0.0351 (9)	0.0254 (10)	0.0332 (11)	0.0012 (7)	0.0052 (8)	−0.0028 (8)
C6	0.0645 (14)	0.0402 (14)	0.0485 (14)	−0.0178 (11)	0.0124 (11)	0.0030 (11)
C7	0.0533 (12)	0.0334 (12)	0.0411 (13)	−0.0091 (9)	0.0131 (10)	0.0072 (10)
C8	0.0410 (10)	0.0241 (10)	0.0244 (10)	0.0048 (8)	0.0018 (8)	−0.0007 (8)
C9	0.0429 (10)	0.0282 (11)	0.0260 (10)	0.0008 (8)	0.0050 (8)	−0.0006 (8)
C10	0.0376 (9)	0.0250 (10)	0.0245 (10)	0.0007 (7)	0.0057 (8)	−0.0035 (8)
C11	0.0410 (10)	0.0352 (12)	0.0306 (11)	0.0053 (8)	0.0021 (8)	0.0019 (9)
C12	0.0408 (11)	0.0527 (14)	0.0421 (13)	0.0134 (10)	0.0028 (9)	−0.0025 (11)
C13	0.0406 (11)	0.0635 (16)	0.0485 (14)	−0.0003 (11)	0.0164 (10)	−0.0020 (12)
C14	0.0511 (12)	0.0486 (14)	0.0379 (13)	−0.0054 (10)	0.0161 (10)	0.0047 (11)
C31	0.0577 (12)	0.0270 (11)	0.0279 (11)	0.0004 (9)	0.0080 (9)	−0.0012 (9)
C32	0.0497 (11)	0.0251 (11)	0.0261 (10)	−0.0015 (8)	0.0012 (8)	−0.0048 (8)
C33	0.0483 (11)	0.0365 (13)	0.0472 (13)	0.0031 (9)	0.0014 (10)	−0.0099 (10)
C34	0.0533 (13)	0.0669 (15)	0.0663 (17)	−0.0117 (11)	0.0217 (12)	−0.0200 (13)
C35	0.0841 (18)	0.0504 (13)	0.0619 (17)	−0.0197 (11)	0.0385 (14)	−0.0100 (12)
C36	0.0818 (17)	0.0306 (12)	0.0455 (14)	0.0039 (11)	0.0211 (12)	0.0050 (10)
C37	0.0485 (11)	0.0317 (12)	0.0349 (12)	0.0026 (9)	0.0105 (9)	−0.0019 (9)
C38	0.143 (4)	0.081 (3)	0.164 (4)	−0.046 (2)	0.093 (3)	0.000 (3)
N1	0.0517 (10)	0.0333 (10)	0.0308 (10)	−0.0021 (8)	0.0061 (8)	−0.0050 (8)
N2	0.1040 (16)	0.0329 (11)	0.0321 (11)	−0.0126 (10)	0.0243 (10)	−0.0022 (8)
N3	0.0464 (9)	0.0267 (9)	0.0267 (9)	0.0032 (7)	0.0049 (7)	0.0074 (7)
N4	0.0481 (9)	0.0259 (9)	0.0284 (9)	−0.0050 (7)	0.0074 (7)	0.0020 (7)
N5	0.0548 (10)	0.0327 (10)	0.0348 (10)	−0.0114 (8)	0.0060 (8)	−0.0036 (8)
O1	0.0814 (11)	0.0412 (9)	0.0393 (9)	−0.0214 (8)	0.0080 (8)	−0.0155 (7)
O2	0.0767 (11)	0.0408 (9)	0.0305 (8)	−0.0099 (8)	0.0104 (7)	−0.0016 (7)
O3	0.0428 (7)	0.0396 (9)	0.0387 (9)	0.0142 (6)	0.0021 (6)	0.0049 (6)
O4	0.1371 (17)	0.0322 (9)	0.0391 (10)	−0.0172 (10)	0.0359 (10)	−0.0120 (7)
Br1	0.05198 (16)	0.0981 (3)	0.0821 (3)	0.03710 (14)	0.00837 (14)	0.01124 (17)

### Geometric parameters (Å, º)

**Table:**

C1—N1	1.378 (3)	C12—C13	1.379 (3)
C1—C5	1.383 (3)	C12—Br1	1.904 (2)
C1—C2	1.523 (3)	C13—C14	1.392 (3)
C2—C10	1.523 (2)	C13—H13	0.9300
C2—C3	1.544 (3)	C14—H14	0.9300
C2—C8	1.564 (3)	C31—O4	1.257 (2)
C3—C4	1.409 (3)	C31—C32	1.502 (3)
C3—C31	1.418 (3)	C32—C33	1.374 (3)
C4—N2	1.324 (3)	C32—C37	1.385 (3)
C4—N4	1.361 (3)	C33—C34	1.388 (3)
C5—N5	1.314 (2)	C33—H33	0.9300
C5—N4	1.365 (3)	C34—C35	1.379 (4)
C6—N5	1.442 (3)	C34—H34	0.9300
C6—C7	1.508 (3)	C35—C36	1.379 (4)
C6—H6A	0.9700	C35—C38	1.525 (4)
C6—H6B	0.9700	C36—C37	1.381 (3)
C7—N4	1.472 (2)	C36—H36	0.9300
C7—H7A	0.9700	C37—H37	0.9300
C7—H7B	0.9700	C38—H38A	0.9600
C8—O3	1.225 (2)	C38—H38B	0.9600
C8—N3	1.341 (2)	C38—H38C	0.9600
C9—C14	1.385 (3)	N1—O2	1.250 (2)
C9—C10	1.388 (3)	N1—O1	1.254 (2)
C9—N3	1.391 (2)	N2—H2A	0.8600
C10—C11	1.378 (3)	N2—H2B	0.8600
C11—C12	1.380 (3)	N3—H3	0.8600
C11—H11	0.9300	N5—H5	0.8600
			
N1—C1—C5	118.64 (17)	C14—C13—H13	120.0
N1—C1—C2	118.86 (16)	C9—C14—C13	117.5 (2)
C5—C1—C2	122.03 (16)	C9—C14—H14	121.2
C1—C2—C10	111.65 (14)	C13—C14—H14	121.2
C1—C2—C3	110.57 (14)	O4—C31—C3	122.18 (18)
C10—C2—C3	113.94 (15)	O4—C31—C32	113.15 (17)
C1—C2—C8	110.53 (15)	C3—C31—C32	124.64 (17)
C10—C2—C8	100.28 (14)	C33—C32—C37	118.93 (19)
C3—C2—C8	109.43 (14)	C33—C32—C31	120.95 (19)
C4—C3—C31	118.04 (17)	C37—C32—C31	119.27 (18)
C4—C3—C2	119.66 (16)	C32—C33—C34	120.1 (2)
C31—C3—C2	122.30 (16)	C32—C33—H33	120.0
N2—C4—N4	115.38 (18)	C34—C33—H33	120.0
N2—C4—C3	123.41 (19)	C35—C34—C33	121.3 (2)
N4—C4—C3	121.21 (17)	C35—C34—H34	119.4
N5—C5—N4	109.02 (17)	C33—C34—H34	119.4
N5—C5—C1	130.78 (18)	C36—C35—C34	118.3 (2)
N4—C5—C1	120.20 (17)	C36—C35—C38	119.8 (3)
N5—C6—C7	103.37 (16)	C34—C35—C38	121.9 (3)
N5—C6—H6A	111.1	C35—C36—C37	120.9 (2)
C7—C6—H6A	111.1	C35—C36—H36	119.6
N5—C6—H6B	111.1	C37—C36—H36	119.6
C7—C6—H6B	111.1	C36—C37—C32	120.5 (2)
H6A—C6—H6B	109.1	C36—C37—H37	119.7
N4—C7—C6	102.60 (16)	C32—C37—H37	119.7
N4—C7—H7A	111.2	C35—C38—H38A	109.5
C6—C7—H7A	111.2	C35—C38—H38B	109.5
N4—C7—H7B	111.2	H38A—C38—H38B	109.5
C6—C7—H7B	111.2	C35—C38—H38C	109.5
H7A—C7—H7B	109.2	H38A—C38—H38C	109.5
O3—C8—N3	127.15 (18)	H38B—C38—H38C	109.5
O3—C8—C2	124.20 (17)	O2—N1—O1	120.52 (17)
N3—C8—C2	108.65 (15)	O2—N1—C1	118.71 (16)
C14—C9—C10	121.71 (18)	O1—N1—C1	120.76 (17)
C14—C9—N3	128.25 (18)	C4—N2—H2A	120.0
C10—C9—N3	110.03 (16)	C4—N2—H2B	120.0
C11—C10—C9	120.72 (17)	H2A—N2—H2B	120.0
C11—C10—C2	130.31 (17)	C8—N3—C9	112.00 (15)
C9—C10—C2	108.97 (15)	C8—N3—H3	124.0
C10—C11—C12	117.34 (19)	C9—N3—H3	124.0
C10—C11—H11	121.3	C4—N4—C5	122.77 (16)
C12—C11—H11	121.3	C4—N4—C7	125.06 (17)
C13—C12—C11	122.7 (2)	C5—N4—C7	110.57 (16)
C13—C12—Br1	118.99 (16)	C5—N5—C6	113.04 (17)
C11—C12—Br1	118.32 (17)	C5—N5—H5	123.5
C12—C13—C14	119.99 (19)	C6—N5—H5	123.5
C12—C13—H13	120.0		
			
N1—C1—C2—C10	−62.7 (2)	C10—C9—C14—C13	−0.9 (3)
C5—C1—C2—C10	109.30 (19)	N3—C9—C14—C13	178.1 (2)
N1—C1—C2—C3	169.32 (16)	C12—C13—C14—C9	0.4 (3)
C5—C1—C2—C3	−18.7 (2)	C4—C3—C31—O4	−8.1 (3)
N1—C1—C2—C8	48.0 (2)	C2—C3—C31—O4	172.2 (2)
C5—C1—C2—C8	−139.99 (18)	C4—C3—C31—C32	169.99 (18)
C1—C2—C3—C4	15.3 (2)	C2—C3—C31—C32	−9.7 (3)
C10—C2—C3—C4	−111.41 (19)	O4—C31—C32—C33	102.3 (2)
C8—C2—C3—C4	137.27 (17)	C3—C31—C32—C33	−75.9 (3)
C1—C2—C3—C31	−164.99 (17)	O4—C31—C32—C37	−67.0 (3)
C10—C2—C3—C31	68.3 (2)	C3—C31—C32—C37	114.8 (2)
C8—C2—C3—C31	−43.0 (2)	C37—C32—C33—C34	−1.0 (3)
C31—C3—C4—N2	−0.7 (3)	C31—C32—C33—C34	−170.3 (2)
C2—C3—C4—N2	178.98 (19)	C32—C33—C34—C35	0.3 (4)
C31—C3—C4—N4	179.52 (18)	C33—C34—C35—C36	0.2 (4)
C2—C3—C4—N4	−0.8 (3)	C33—C34—C35—C38	178.4 (3)
N1—C1—C5—N5	−1.1 (3)	C34—C35—C36—C37	0.1 (4)
C2—C1—C5—N5	−173.18 (19)	C38—C35—C36—C37	−178.1 (3)
N1—C1—C5—N4	179.15 (16)	C35—C36—C37—C32	−0.9 (3)
C2—C1—C5—N4	7.1 (3)	C33—C32—C37—C36	1.3 (3)
N5—C6—C7—N4	−11.3 (2)	C31—C32—C37—C36	170.8 (2)
C1—C2—C8—O3	61.5 (2)	C5—C1—N1—O2	−176.85 (18)
C10—C2—C8—O3	179.41 (18)	C2—C1—N1—O2	−4.6 (3)
C3—C2—C8—O3	−60.5 (2)	C5—C1—N1—O1	2.4 (3)
C1—C2—C8—N3	−119.69 (16)	C2—C1—N1—O1	174.70 (17)
C10—C2—C8—N3	−1.77 (19)	O3—C8—N3—C9	−178.42 (19)
C3—C2—C8—N3	118.31 (16)	C2—C8—N3—C9	2.8 (2)
C14—C9—C10—C11	0.7 (3)	C14—C9—N3—C8	178.2 (2)
N3—C9—C10—C11	−178.48 (17)	C10—C9—N3—C8	−2.7 (2)
C14—C9—C10—C2	−179.42 (18)	N2—C4—N4—C5	166.80 (18)
N3—C9—C10—C2	1.4 (2)	C3—C4—N4—C5	−13.5 (3)
C1—C2—C10—C11	−62.8 (3)	N2—C4—N4—C7	2.6 (3)
C3—C2—C10—C11	63.3 (3)	C3—C4—N4—C7	−177.68 (18)
C8—C2—C10—C11	−179.93 (19)	N5—C5—N4—C4	−169.47 (17)
C1—C2—C10—C9	117.27 (17)	C1—C5—N4—C4	10.3 (3)
C3—C2—C10—C9	−116.59 (17)	N5—C5—N4—C7	−3.2 (2)
C8—C2—C10—C9	0.18 (19)	C1—C5—N4—C7	176.55 (17)
C9—C10—C11—C12	0.1 (3)	C6—C7—N4—C4	175.24 (19)
C2—C10—C11—C12	−179.74 (19)	C6—C7—N4—C5	9.4 (2)
C10—C11—C12—C13	−0.7 (3)	N4—C5—N5—C6	−5.0 (2)
C10—C11—C12—Br1	179.96 (14)	C1—C5—N5—C6	175.2 (2)
C11—C12—C13—C14	0.4 (4)	C7—C6—N5—C5	10.7 (2)
Br1—C12—C13—C14	179.77 (17)		

### Hydrogen-bond geometry (Å, º)

Cg1 is the centroid of the C32–C37 ring.

**Table:**

*D*—H···*A*	*D*—H	H···*A*	*D*···*A*	*D*—H···*A*
N5—H5···O1	0.86	2.09	2.608 (2)	118
N2—H2*B*···O4	0.86	1.87	2.518 (2)	131
N3—H3···O4^i^	0.86	1.95	2.792 (2)	168
N5—H5···O3^ii^	0.86	2.36	2.961 (2)	127
C7—H7*A*···O3^iii^	0.97	2.54	3.342 (3)	140
C33—H33···Br1^iv^	0.93	2.91	3.675 (2)	141
C14—H14···*Cg*1^i^	0.93	2.83	3.553 (2)	135

Symmetry codes: (i) *x*, −*y*+1/2, *z*−1/2; (ii) −*x*, *y*−1/2, −*z*+1/2; (iii) −*x*, −*y*, −*z*+1; (iv) −*x*+1, −*y*, −*z*+1.
